# Multi-Omics Analysis Reveals That *SlERF.D6* Synergistically Regulates SGAs and Fruit Development

**DOI:** 10.3389/fpls.2022.860577

**Published:** 2022-04-08

**Authors:** Hao Guo, Mengdi Mao, Yuan Deng, Lisong Sun, Ridong Chen, Peng Cao, Jun Lai, Yueran Zhang, Chao Wang, Chun Li, Yiran Li, Qunhang Bai, Tingting Tan, Jun Yang, Shouchuang Wang

**Affiliations:** ^1^College of Tropical Crops, Hainan University, Haikou, China; ^2^Hainan Yazhou Bay Seed Laboratory, Sanya Nanfan Research Institute of Hainan University, Sanya, China; ^3^School of Life and Pharmaceutical Sciences, Hainan University, Haikou, China

**Keywords:** tomato, AP2/ERF, mGWAS, *SlERF.D6*, fruit development, steroidal glycoalkaloids

## Abstract

Steroidal glycoalkaloids (SGAs) are cholesterol-derived molecules that contribute to the pathogen defense in tomato but are toxic and considered to be antinutritional compounds to humans. APETALA2/Ethylene Responsive Factor (AP2/ERF) family transcription factors (TFs) play an indispensable role in various biological processes, such as plant growth and development, fruit ripening, biotic and abiotic stresses responses, and SGA biosynthesis. In this study, we identified 176 *AP2/ERF* genes that were domesticated or improved *SlAP2/ERF* in the tomato variome (*Solanum lycopersicum*) within either domestication or improvement sweeps, respectively. According to the RNA-sequencing data, 93 of the *ERF* genes with high transcriptional level (Transcripts Per Million, TPM > 1) belong to six clusters. Weighted gene co-expression network analysis (WGCNA) and metabolite-based genome-wide association study (mGWAS) analyses revealed that the expression level of the *Solyc04g071770* (*SlERF.D6*) gene in the cluster six gradually increased as the fruit matured. Transient transformation verified that the overexpression of *SlERF.D6* significantly promoted fruit ripening and regulated the expression of multiple genes in the SGA synthesis pathway, thereby affecting the SGA content of the fruit. Virus-induced gene silencing (VIGS) showed that the silencing of *SlERF.D6* delayed fruit ripening and influenced the content of SGAs. Our data provide new insights into AP2/ERF TFs in tomato, offer a candidate TF for fruit development and steroidal glycoalkaloids, and provide new resources for tomato breeding and improvement.

## Introduction

Tomato (*Solanum lycopersicum*) originated in the Andean region of South America. It is a model plant for genetic improvement and biological research system for Solanaceae crops, such as potato, pepper, and eggplant (Ranc et al., 2008). It is the most valuable fruit and vegetable crop in the world and contributes significantly to the amount of nutrients required to feed the human population (Klee and Tieman, 2018). Solanaceae plants produce a specific metabolite called steroidal glycoalkaloids (SGAs), the glycosylated forms of steroidal alkaloids (SAs), which help the plants protect themselves from bacteria, fungi, viruses, and certain insects during growth. For humans, SGAs are antinutritional compound that can either cause toxic reactions by inhibiting the cholinesterase activity in neurons or rupture lipid membranes through sterols (Aziz et al., 2012; Dzakovich et al., 2020; Hennessy et al., 2020).

In recent years, a series of research progresses have been made in the structural genes and transcription factors (TFs) of SGAs metabolic synthesis (Itkin et al., 2013). Glycoalkaloid metabolism 1, 17, 18, and 2 (GAME1, GAME17, GAME18, and GAME2) are glycosyltransferases, mainly performing multi-step glycosylation in the SGAs metabolic pathway. In addition to this, some P450s (GAME7, GAME8, GAME6, and GAME4), a dioxygenase (GAME11), and a transaminase protein (GAME12) are involved in the hydroxylation and oxidation of the cholesterol skeleton. Then, GAME5 is involved in the production of esculeoside A (Itkin et al., 2013). In addition, some TFs are involved in the regulation of SGAs. *GAME9*, an APETALA2/Ethylene Responsive Factor (AP2/ERF), plays a synergistic role in regulating the SGAs synthesis pathway. A 1 bp substitution in *GAME9* was significantly associated with a high SGA content. It also affected the binding capacity of *GAME17* promoter, a core SGA biosynthetic gene, with *GAME9* (Cárdenas et al., 2016; Yu et al., 2020). This indicates that AP2/ERF plays a key role in the biosynthesis of SGAs.

In general, plant TFs play vital roles in plant growth, development, and environmental stress responses (Shu et al., 2016). AP2/ERF superfamily is one of the largest groups of TFs (Jofuku et al., 1994; Wessler, 2005), and the AP2/ERF proteins are involved in biological processes related to the plant growth and response to the biotic and abiotic stress in many species (Sharabi-Schwager et al., 2010; Qi et al., 2011). *OsEATB*, an *AP2/ERF* superfamily gene in *Oryza sativa* subsp., reduces rice plant height and panicle length at maturity by downregulating a gibberellin biosynthetic gene in the presence of ethylene (Qi et al., 2011). The *DREB/CBF2* were shown to be involved in plant cold stress response, and the overexpression of *CBF2* in *Arabidopsis* delayed plant growth, flowering, and senescence (Sharabi-Schwager et al., 2010). In tomato, SlAP2/ERFs have been shown to affect many biological functions, such as the regulation of growth of various phytohormones (i.e., cytokinin, ethylene, and methyl jasmonate) (Chung et al., 2010; Liu et al., 2013; Li et al., 2017; Sun et al., 2018; Lin et al., 2020; Zhang et al., 2021). Although Liu et al. (2016) updated the number of *AP2/ERF* genes to 146, there are still many potential *AP2/ERF* genes unknown. With the development of sequencing technology and multi-omics, it is crucial to re-identify *AP2/ERF* genes using new tomato genome data, and to analyze the function of *AP2/ERF* genes, such as the regulation of metabolism, in combination with multi-omics.

In this study, we re-identified the members of the *SlAP2/ERF* gene family using NGS tomato genome data, and the number was updated to 176. Additionally, we analyzed the characteristics and the evolutionary and phylogenetic relationships between these genes. By combining transcriptomic, metabolomic, and genomic, we had inferred and validated the functions of *AP2/ERF* genes, which revealed that some of them were selected during tomato breeding. Using both weighted gene co-expression network analysis (WGCNA) and metabolite-based genome-wide association study (mGWAS), we found that *SlERF.D6* coordinates the development of tomato fruit and the metabolic synthesis of SGAs. In this study, we identified the role of *SlERF.D6* in fruit ripening and the synthesis of SGAs to provide a theoretical basis for cultivating better tomato varieties.

## Materials and Methods

### Plant Materials and Sampling

The variety Micro-Tom was used as wild-type (WT) for the genetic materials in this study. Tomato plants were grown in a greenhouse at 24°C under a 16-h-light and 8-h-dark cycle at Hainan University, Haikou, China. RNA-sequencing (RNA-seq) samples were harvested at different stages during the fruit development of tomato. The skin of fruits was collected from days post-germination (DPG) at immature green (IMG in 60 DPG), mature green (MG in 80 DPG), breaker (Br in 90 DPG), and ripe fruit (Ripe in 100 DPG) stages, four periods with three biological replicates were collected. All samples were collected before the greenhouse lights were turned off at approximately 7:00–9:00 p.m. Samples were collected from the plants, immediately placed in liquid nitrogen.

### Identification of Tomato *AP2/ERF* Gene Family

Tomato ITAG3.2 genome data were obtained from Sol Genomics Network.^1^ The Hidden Markov model (HMM) data of AP2 domain were downloaded from Pfam database^2^ with domain number PF00847. We scanned the tomato genome by HMMER 3.2.1 with default parameters. The presence of AP2 sequences was confirmed by using Pfam (see text footnote 2) and Simple Modular Architecture Research Tool (SMART)^3^ to further check for all candidate genes that may contain AP2/ERF structural domains based on HMMER software results. We expanded ERF gene numbering based on the naming system provided by Liu et al. (2016). For genes from other subfamilies, we numbered them sequentially according to their chromosomal locations. The sequence length, molecular weight, and isoelectric point (pI) of the identified AP2/ERF family proteins were obtained using the tools on the ExPasy website.^4^ The predicted subcellular localization data for all *AP2/ERF* genes were obtained *via* the GenScript website.^5^

### RNA Sequencing and Data Analysis

We collected tomato skin from the four key fruit development periods (IMG, MG, Br, and Ripe) for RNA-seq in this study. Total RNA-seq of all samples was extracted by TRIzol method. Besides, the transcriptome libraries were constructed by using an Illumina TruSeq RNA kit according to the manufacturer’s recommendations. Sequencing was performed using an Illumina HiSeq X Ten system. RNA-seq data from each sample were obtained for three biological replicates. The quality of RNA-seq data was examined using Fastp (0.20.1), and adapter sequences and low-quality reads were filtered by Fastp with default parameters. Reads were mapped to *S. lycopersicum* reference genome (ITAG 3.2) using Hisat2 (Kim et al., 2019). FeatureCounts (v2.0.1) was used to estimate the expression levels of all genes (Liao et al., 2019), with the command: featureCounts −T −p −t exon −g gene_id −a. Transcripts Per Million (TPM) was used to normalize the expression levels of all samples. Different expression genes (DEGs) were identified using DESeq R package (Anders and Huber, 2010), with the threshold for fold change ≥ 2 and the significance level <0.01.

### Multiple Sequence Alignment and Phylogenetic Tree Construction

Multiple sequence alignment of the AP2 structural domain of the AP2/ERF protein from *Arabidopsis thaliana* (Nakano et al., 2006) was performed using the software Gblocks to obtain the conserved structural sequence.^6^ The alignment was used through Molecular Evolutionary Genetics Analysis (MEGA) X using the ClustalW method with default parameters (Kumar et al., 2018). Neighbor-Joining (NJ) tree construction was performed using MEGA X with the Poisson model, pairwise deletion with 1,000 bootstrap replications.

### Chromosomal Distribution, Gene Duplication, and Synteny Analysis

All *SlAP2/ERFs* distribution information on chromosomes were based on the tomato genome database and visualized by TBtools. The results visualized using the Circos software. Tandem duplication and segment duplication were explored by using the Multiple Collinearity Scan toolkit X (MCScanX) (Wang et al., 2012). Synteny analysis was performed within the tomato genome and between multiple species also using MCScanX, and the genome data of other species were downloaded from the ensemble website.^7^

### Weighted Gene Co-expression Network Analysis and Co-expression Analysis

Weighted gene co-expression network analysis was performed to generate the gene expression network based on the correlation patterns among all high expression genes (Fragments Per Kilobase per Million, FPKM > = 1). The construction of a weighted co-expression network needs the soft-thresholding powers β, which were calculated by the pick soft threshold function of the R Package (WGCNA version 1.70-3) (Langfelder and Horvath, 2008). We chose the power 10 as it is the lowest power for which the scale-free topology fit index curve flattens out reaching a high value (above 0.9). The network of modules of co-expressed genes (edge weight >0.10) was represented by Cytoscape 3.7.1 (Otasek et al., 2019). The expression levels of *SlAP2/ERF* genes and SGA pathway genes generated in this study were used for co-expression analysis, and Pearson’s correlation coefficients (PCC) were used to measure the co-expression relationships between genes.

### Genome-Wide Association Analysis and Linkage Disequilibrium

Whole genome sequencing (WGS) raw data were used from previous studies (Lin et al., 2014; Zhu et al., 2018). A total of 1,476,413 single nucleotide polymorphisms (SNPs) with minor allele frequency (MAF) >5% and missing rate <10% from 349 accessions were used as genotypic data. The phenotypic data used the relative content of esculeoside A from our previous study (Zhu et al., 2018), and then, we performed the genome-wide association analysis. Efficient Mixed-Model Association eXpedited (EMMAX) software was used to test the associated relationships between genotype and phenotype (Kang et al., 2010), and population structure was modeled as a random effect in Efficient Mixed-Model Association (EMMA) using Admixture software, the kinship (K) matrix was calculated by emmax-kin, the parameter is emmax-kin −v −h −s −d 10. The effective number of independent SNPs was calculated using Genetic type 1 Error Calculator (GEC) software (Li et al., 2012). The calculated genome-wide significance thresholds, based on a nominal level of 0.05, were *p* = 4.19 × 10^––7^ for the whole population. Linkage disequilibrium (LD) was calculated based on the *R*^2^ value by Plink (v1.9).^8^

### Vector Construction

The full-length coding sequence (CDS) of *SlERF.D6* was obtained from the Sol Genomics Network database (see text footnote 1) and amplified from the cDNA of Micro-Tom leaves, then it was introduced into the entry vector pDONR207 and the destination vectors using the Gateway Cloning Technology (Curtis and Grossniklaus, 2003). Virus-induced gene silencing (VIGS) specific fragment (300 bp) was selected through the VIGS tool in the Sol Genomics Network database. After amplification and ligation to TRV2 vector (Orzaez et al., 2006), the plasmid TRV2-*SlERF.D6* can be introduced into *Agrobacterium tumefaciens* strain GV3101 (Weidi, Shanghai, China).

### Expression Analysis by Quantitative Real-Time PCR

Tomato fruits were harvested at Br5 and the fruit skin was ground into a fine powder in liquid nitrogen. Total RNAs were extracted by using a RNAprep Pure Plant Plus Kit (Polysaccharides & Polyphenolics-rich) (TIANGEN, Beijing, China). The total RNA (2 μg) was reverse-transcribed to cDNA using the TransScript^®^ One-Step RT-PCR SuperMix (TransGen, Beijing, China) according to the manufacturer’s instructions at 42°C for 30 min and 85°C for 10 s. A quantitative real-time PCR (qRT-PCR) was performed using the SYBR Premix ExTaq (Vazyme, Nanjing, China) by Quantstudio™ Real-Time PCR Software. Tomato *ubiquitin* gene (*Solyc01g056940*) was used for normalization, the relative expression level of each gene was calculated using the 2^–ΔΔ*Ct*^ method (Livak and Schmittgen, 2001). The primer pairs for qRT-PCR were designed using the Primer 5.0 and blasted at National Center for Biotechnology Information (NCBI) database to ensure primer specific. Three biological replicates were performed for each qRT-PCR analysis. Primers are listed in Supplementary Table 7.

### Metabolite Profiling

For the measurement of SGAs, tomato fruits were harvested and half of the fruit pericarp was freeze-dried. Metabolite extraction and profiling were performed in the same way as described in the previous study (Zhu et al., 2018).

### Transient Transformation

The plasmid *TRV2*-*SlERF.D6*, TRV2 and TRV1 were introduced into *A. tumefaciens* strain, then injected into MG fruit with the TRV1 vector combined with TRV2 or *TRV2*-*SlERF.D6* plasmid, the TRV2 vector was used as control. The injection was performed as described previously (Orzaez et al., 2006; Fantini and Giuliano, 2016). The skin samples were collected 10 days after injection and immediately placed in liquid nitrogen. The vector *pBin18-E8-SlERF.D6* used for transient overexpression was introduced into *Agrobacterium* and then injected into the tomato MG fruit in the same way, as the pBin18-E8 vector was used as control (Orzaez and Granell, 2009; Zhao and Zhao, 2009). Samples (skin) were taken into liquid nitrogen after 8 days.

### Yeast One-Hybrid Assay

The promoter fragments of *GAME2, 5, 12*, and *18* were amplified and introduced into pHis2 to construct bait vectors (BD) and the CDS of *SlERF.D6* was introduced into pGADT7 to construct a prey vector (AD). All plasmids were transformed into Y187 Chemically Competent Cell (TOLOBIO, Shanghai, China) and cultured on SD/-Leu-Trp at 30°C for 2 days. The positive yeast strains were picked and diluted with sterile water to four concentrations. These suspensions were spotted on an SD/−Leu-Trp-His medium and incubated at 30°C for 3 days.

### Dual-Luciferase Transient Expression Assay

The CDS of *SlERF.D6* was introduced into the pEAQ-HT-DEST2 vector acting as an effector vector and the promoter fragments of *GAME12* were amplified and introduced into the modified pH2GW7 vector containing the firefly luciferase (LUC) gene and the Renilla luciferase (REN) gene acting as a reporter vector. All plasmids were transferred into *A. tumefaciens* strain GV3101 (Weidi, Shanghai, China) and then injected into the *Nicotiana benthamiana* leaves. The activities of LUC and REN luciferase were measured using the Dual-Luciferase Assay Kit (Promega) according to the instruction manual. The relative reporter gene expression levels were expressed as the ratio of firefly LUC to REN luciferase (LUC/REN). Six biological replicates for each sample were performed.

## Results

### Identification of the *SlAP2/ERF* Genes

By conducting an integrative analysis on the tomato ITAG3.2 genome and multiple other databases, we identified a total of 176 *AP2/ERF* genes in tomato. Multiple protein sequence alignment and phylogenetic analysis revealed that all the AP2/ERF proteins in *A. thaliana* (127) and tomato (176) are mostly divided into three major clades (Supplementary Figure 1). Based on the above phylogenetic tree and the number of AP2 domains, 146, 26, and 3 of the genes that were identified as belonging to the ERF, AP2, and RAV subfamilies, respectively. In addition, a single gene, *Solyc09g059510*, that was homologous to *At4g13040* (Giri et al., 2014) belonged to the Soloist subfamily. We explored the size of sequence, predicted subcellular localization, molecular weight, pI, and intron number of *SlAP2/ERFs* (Supplementary Tables 1, 2). In summary, new *SlERFs* were identified and their characteristics and structures herein were statistically determined.

**FIGURE 1:**
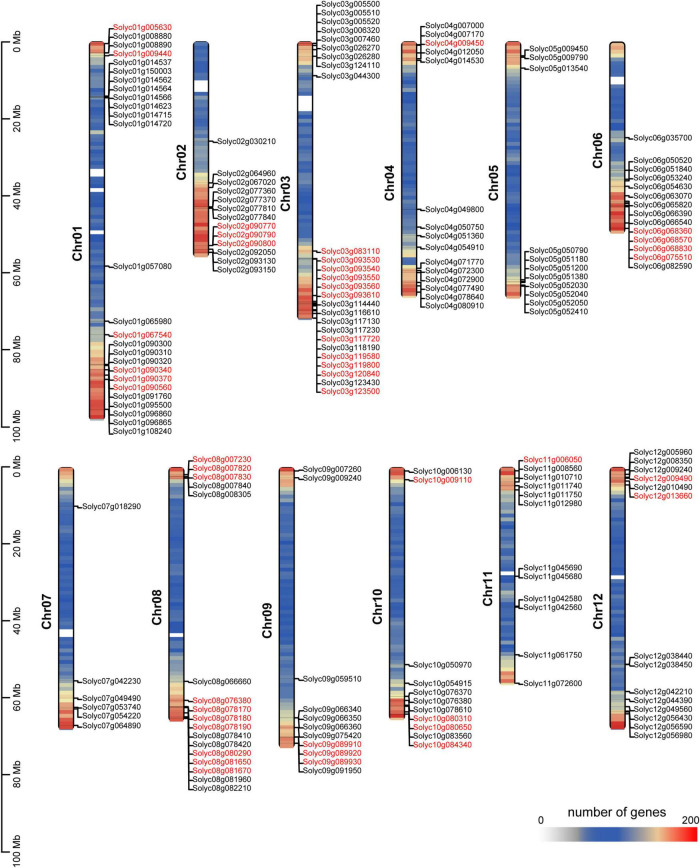
Distribution of the identified 176 *SlAP2/ERF* genes across the tomato genome. The density of genes on 12 chromosomes are shown. All tomato chromosomes are drawn to scale based on their actual physical lengths. The genes with red color represent that they locate on domestication or improvement sweeps. The color of chromosomes represents the number of genes.

Tomato breeding has undergone the two stages of domestication (PIM-CER, i.e., *Solanum pimpinellifolium* to S. *lycopersicum* var. *cerasiforme*) and improvement (CER-BIG, i.e., *S. lycopersicum* var. *cerasiforme* to *S. lycopersicum*). Fruit weight is artificially selected upon in modern tomato breeding programs, possibly leading to the selection of *SlAP2/ERF* genes (Lin et al., 2014). To characterize the role of these genes in domestication or improvement, we integrated the identification results of domestication/improvement sweeps in our previous study and the distribution of *SlAP2/ERF* genes (Zhu et al., 2018). If genes were located in the domestication or improvement sweeps, it means that these genes may regulate certain traits, such as fruit size that were selected by ancient farmers. We call these genes “domesticated” or “improved” genes and marked all domesticated or improved *SlAP2/ERF* genes on chromosomes (Figure 1). The results showed that one *RAV* gene and eight (more than 30%) *AP2* genes were selected by humans. In total, 36 genes in the ERF subfamily located in the selection sweep, such as *Solyc01g090340*, *Solyc10g009110*, and *Solyc03g119580* which have been reported to regulate the SGA synthesis, fruit growth, and development (Pirrello et al., 2012; Shi et al., 2012; Thagun et al., 2016). These data indicate that the *SlAP2/ERF* genes play a crucial role in tomato development and were introduced during the selection for fruit weight.

### *SlERF* Expression Regulates Fruit Ripening and Steroidal Glycoalkaloid Accumulation

**FIGURE 2:**
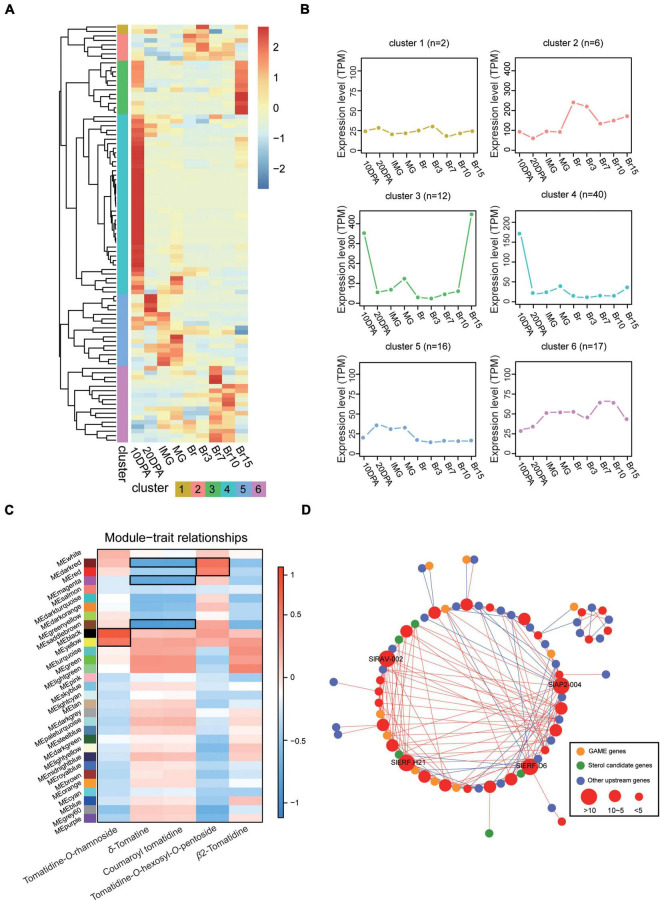
RNA-seq analysis of *ERF* genes during fruit development. **(A,B)** Expression pattern of the *ERF* genes during the fruit development of tomato. The gradient color (blue to red) in the heatmap represents the transcription level after normalization, and other colors represent different clusters. **(C)** Module-sample association. Each row corresponds to a module with different colors, labeled with a color as in Supplementary Figure 9. The color of each cell at the row–column intersection indicates the correlation coefficient between the module and the steroidal glycoalkaloids (SGAs) content, and the modules with black border represented that they are highly correlated with SGAs. **(D)** Analysis of co-expression between *SlAP2/ERF* genes and other SGA related genes. Different colored circles represent different genes, red is the AP2/ERF identified in this study, and different circle sizes are the number of their high correlation with other genes.

To further characterize the role of *SlERFs* in tomato, a phylogenetic tree was constructed with *A. thaliana* ERF proteins using the NJ method. These genes were divided into two major branches with nine groups. They were named *DREB* and *ERF* (Supplementary Figure 3), it is similar to other plants. Then, previously published tomato RNA-seq data collected from developing tomato fruit were used to explore SlERF-related expression patterns (Li et al., 2020). A total of 93 *ERF* genes with high transcription level (TPM > 1 at least one stage) were expressed from 10 days post-anthesis (DPA) to Br15 (days post-breaker stage) (Figure 2A). Hierarchical clustering showed that the *ERF* genes expression was divided into six clusters. During early fruit development, these *ERF* genes were highly expressed in cluster 4. During the late fruit development, they were more abundant in clusters 2, 3, and 6 (Figure 2B). These results imply that highly expressed ERFs play a stronger regulatory role during late fruit development.

In addition, we performed pairwise comparisons on RNA-seq data generated in this study during four key fruit development periods (IMG, MG, Br, and Ripe) to identify the DEGs. Among them, MG possessed the largest number of DEGs (4,803) compared with Ripe and 1,660 DEGs were identified between IMG and Br, which is the minimum number (Supplementary Table 4 and Figure 5). We found 14 genes that were not only significantly differentially expressed during tomato development, but also in an expression cluster we speculated was associated with fruit ripening (Figure 2B). Groups III and VII contained 28.6 and 21.5% of the 14 genes, respectively, which is more than any other group (Supplementary Figure 6). Several of these *ERF* genes, such as *CBF1* and *ERF1a*, reportedly boosted the fruit development (Huang et al., 2004). These results suggest that our approach is appropriate and that some *ERF* genes with currently unknown functions may greatly influence the fruit development and ripening.

To understand whether other *ERF* genes can regulate SGA biosynthesis during tomato fruit development, the previously published tomato RNA-seq data were analyzed using WGCNA (Li et al., 2020; Supplementary Figures 7–9 and Supplementary Table 5). A total of 31 modules (each module is labeled with a unique color) were produced after filtering out low-transcription level (FPKM < 1) genes. Next, we associated each of the co-expression modules with metabolite concentrations in developing tomato fruits reported by previous studies (Li et al., 2020). Four of the modules were significantly positively correlated with SGA accumulation (PCC > = 0.60, *p* < 0.05), and three modules were significantly negatively correlated with SGA accumulation during tomato fruit development. In one of the modules, the gene expression was both positively and negatively correlated with SGA expression (Figure 2C). This suggests that the genes in these modules regulate SGA biosynthesis. Among them, it was found that the yellow and black modules were significantly positively correlated with Tomatidine-*O*-rhamnoside expression. Furthermore, we constructed the co-expression network between SlAP2/ERF and SGAs pathway-related genes using RNA-seq data by this study. When we looked for *SlAP2/ERF* and SGA pathway genes, we found 10 *GAME* genes, 5 sterol candidate genes, 28 upstream of genes involved in these pathways, and 29 *SlAP2/ERF* genes (PCC > 0.8 or PCC < −0.8 and *p* < 0.05). Furthermore, four *SlAP2/ERF* genes (*SlAP2-004*, *SlRAV-002*, *SlERF.H21*, and *SlERF.D6*) were highly co-expressed with multiple (gene number >10) SGA pathway genes (Figure 2D). This suggests that these four *SlAP2/ERF* genes regulate the expression of other SGA pathway genes which, in turn, regulates the SGAs biosynthesis. Since these four genes were also present in WGCNA modules that were highly associated with SGA pathways, we further speculated that they play important roles in the SGA biosynthesis.

### Use of Multi-Omics to Identify Steroidal Glycoalkaloid Biosynthesis Candidate Genes

**FIGURE 3:**
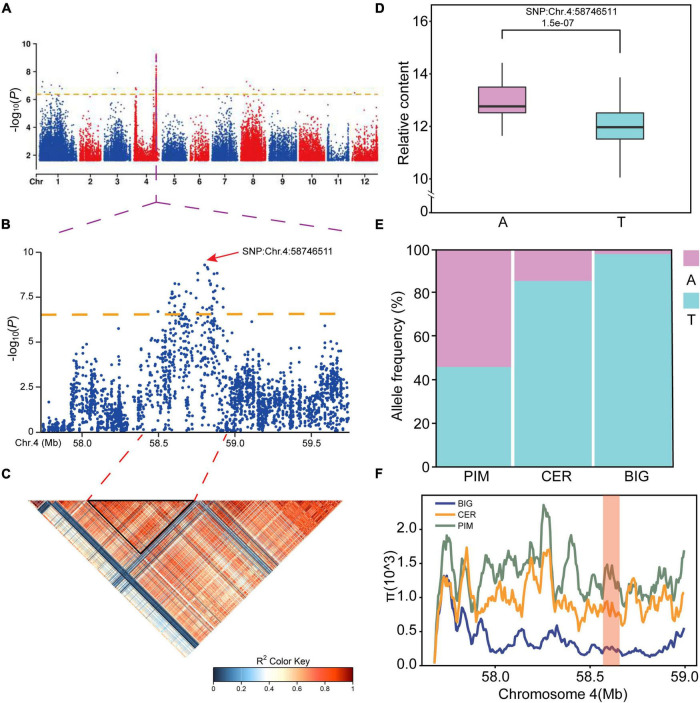
Assignment of the putative causative gene of the natural variation in esculeoside A content. **(A)** Genome-wide association study (GWAS) of esculeoside A content. The orange dashed line represents the significance threshold (*p* = 4.19E–7). Region Manhattan plot **(B)** and linkage disequilibrium (LD) heatmap (bottom) **(C)** surrounding the peak on chromosome 4. **(D)** Box plot for the normalization content by log_2_ of esculeoside A plotted as a haplotype, Chr.4:58746511. **(E)** The allele distribution of varieties containing A or T allele among tomato subpopulations. **(F)** The causative locus is located within a sweep region with much lower nucleotide diversity in BIG than in either CER or PIM. The red column represents the region around *SlERF.D6*.

In plant breeding, genetic gain depends on broad genetic variation (Shi and Lai, 2015). To find the genetic sources of natural variations in SGAs levels during tomato development, metabolite profiling data and high-quality SNPs were obtained from previous studies of 349 *S. lycopersicum* accessions (Supplementary Table 6). To identify the potential genetic basis of natural variations in SGA levels in tomato, mGWAS was performed on accessions displaying substantial differences in SGAs biosynthesis. One *ERF* gene locus, *Solyc04g071770* (*SlERF.D6*), was significantly [*p* = 4.19 × 10^–7^, Mixed Linear Model (MLM)] associated with the accumulation of esculeoside A (Figure 3A). Of note, this metabolite is produced downstream of the SGA biosynthesis pathway.

On the Manhattan plot, the most significant SNP was found at Chr.4:58746511 (Figure 3B). The LD of the SNPs in this region ranged from 57.75 to 59.875 Mb, as shown on the LD heatmap (Figure 3C). This result shows that there is an LD block near the most significant SNP, and *SlERF.D6* (inferred to be a regulator of SGA biosynthesis) is in this block. These results indicate that *SlERF* genes may be responsible for the differences in SGA biosynthesis among the various tomato genotypes.

Further investigation revealed that the lead SNP, Chr.4:58746511, is a non-synonymous T to A mutation found within the coding region of *Solyc04g071770*. The SNP, which is located at the 593 bp position of the gene, converts an amino acid from Val to Glu. Haplotype analysis revealed that the SNP is significantly highly associated with the content of esculeoside A with metabolite ID of SlFM1998 (Figure 3D and Supplementary Table 6). Thus, it could be responsible for the variation observed in the developing tomato fruit. We found that the MAF of this SNP was not uniform among the tomato subpopulations. The frequency of the alternate allele was less than 3% in BIG, about 14.5% in CER, and as high as 54.5% in PIM (Figure 3E), suggesting that this gene was inadvertently selected by ancient farmers. To further understand their selection process, we measured the nucleotide diversity in this region of the genome (Figure 3F). Although it did not reside with a domestication or improvement sweep, a more pronounced difference between PIM, CER, and BIG genomes was found, hinting that it could be a target for future domestication sweeps. Based on these results, we found a natural variation of a gene regulating SGA biosynthesis, and the frequency of SNP was significantly different from wild tomato to cultivars.

### SlERF.D6 Promotes the Ripening of Tomato Fruit

**FIGURE 4:**
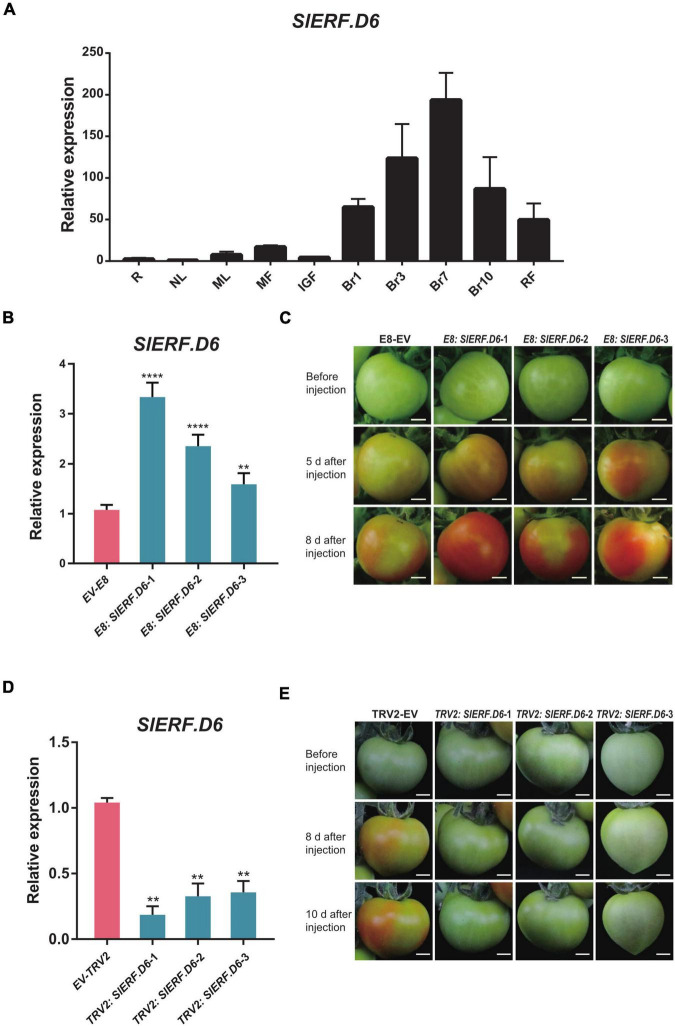
The overexpression of *SlERF.D6* can promote the fruit ripening. **(A)** The expression level of *SlERF.D6* in different tissues and fruits in different periods by quantitative real-time PCR (qRT-PCR). The *X*-axis refers to the different tissues of tomato and the *Y*-axis is the relative expression. R, root; NL, new leaf; ML, mature leaf; MF, mature flower; IGF, immature green fruit; RF, red fruit; Br1, 1 day post breaker; Br3, 3 days post breaker; Br7, 7 days post breaker; Br10, 10 days post breaker; RF, red fruit. **(B)** Expression level of *SlERF.D6* in *E8: SlERF.D6* lines fruit. *X*-axis represents different lines. The empty vector (EV-E8) serves as control. **(C)** Phenotypes of *E8: SlERF.D6* lines fruit after injection. Bars, 0.5 cm. **(D)** Expression level of *SlERF.D6* in *TRV2: SlERF.D6* lines fruit. *X*-axis represents different lines. The empty vector (EV-TRV2) serves as control. **(E)** Phenotypes of *TRV2: SlERF.D6* lines fruit after injection. All the above error bars represent the SD (*n* = 3) (**p < 0.01, ****p < 0.0001; Student’s *t*-test).

To further understand the function of SlERF.D6, a qRT-PCR was performed to characterize the expression pattern of *SlERF.D6* in different tomato tissues and different stages of fruit ripening. The result showed that the expression level gradually increased as fruit ripening progressed, indicating that SlERF.D6 may influence fruit ripen (Figure 4A). To investigate the role of SlERF.D6 in tomato fruit ripening, we performed transient expression in tomato fruit. In recent years, transient expression techniques have been applied to several species of plants (Hamilton et al., 1992; Liu et al., 2010). We first transiently overexpressed SlERF.D6 under the control of a fruit-specific E8 promoter (as shown in Experimental procedures). The qRT-PCR analysis showed that the expression level of *SlERF.D6* was significantly higher in E8: SlERF.D6 tomato fruits than in the control sample (E8-EV). Thus, we successfully obtained three separate *SlERF.D6* transient overexpression lines (Figure 4B and Supplementary Table 8). After injection, we observed that the fruit from the *E8: SlERF.D6* lines split slightly more than the fruit from the control sample after 5 days. However, the color of fruit from the *E8: SlERF.D6* lines were much redder relative to the control sample after 8 days (Figure 4C).

**FIGURE 5:**
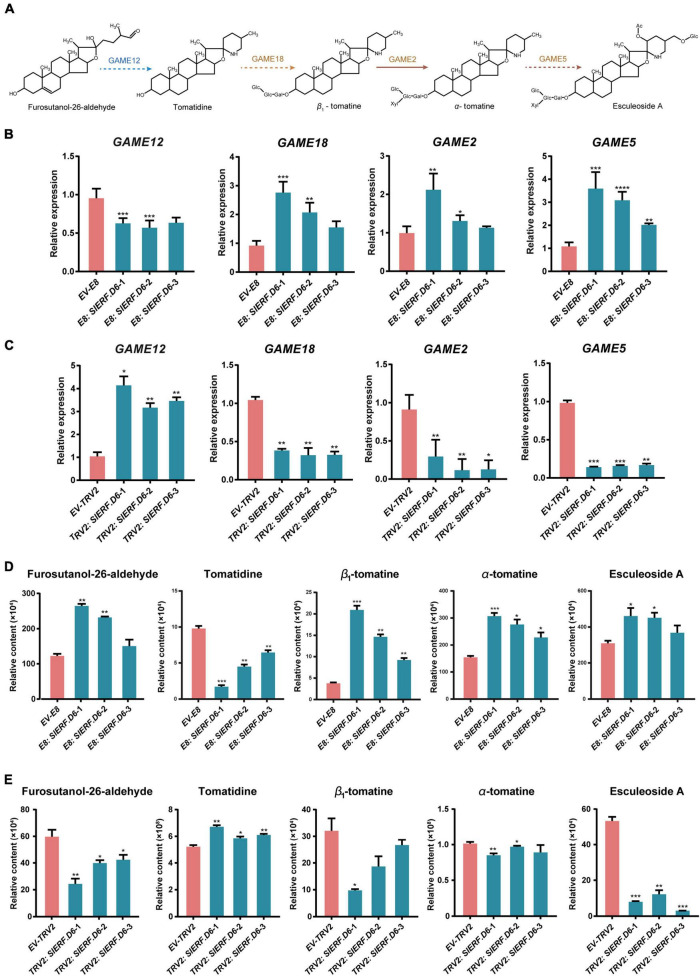
SlERF.D6 influences the content of SGAs in tomato fruit. **(A)** Schematic representation of SGAs biosynthesis and regulation in tomato. **(B)** Expression level of some SGAs pathway genes in *E8: SlERF.D6* lines. *X*-axis represents different lines and the *Y*-axis represents relative expression. **(C)** The expression level of some SGAs pathway genes in *TRV2: SlERF.D6* lines. *X*-axis represents different lines and the *Y*-axis represents relative expression. **(D)** The contents of some related metabolites in *E8: SlERF.D6* lines. The *Y*-axis represents relative content. **(E)** The contents of some related metabolites in *TRV2: SlERF.D6* lines. The *Y*-axis represents relative content. All the above error bars represent the SD (*n* = 3) (*p < 0.05, **p < 0.01, ***p < 0.001, ****p < 0.0001; Student’s *t*-test).

In addition, we performed VIGS to further explore the effect of SlERF.D6 on fruit development, which has been widely used in tomato fruit (Orzaez et al., 2006, 2009; Fantini and Giuliano, 2016). By TRV-mediated gene silencing, we obtained several lines with the reduced expression level of *SlERF.D6* (*TRV2: SlERF.D6*) (Figure 4D and Supplementary Table 8). Besides, its phenotype was opposite to *E8: SlERF.D6*, when the fruit of EV (TRV2) had mostly turned red, *TRV2: SlERF.D6* lines did not yet start to enter the stage of breaker (Figure 4E). These findings indicate that SlERF.D6 had a strong effect on the growth and development of tomato fruits, and that the overexpression of it promoted fruit ripening.

### SlERF.D6 Regulates Steroidal Glycoalkaloids Metabolism During Tomato Fruit Development

To better explore the relationship between SlERF.D6 and SGA metabolism, we measured the transcription level of several SGA biosynthesis-related genes using qRT-PCR analysis (Figures 5A–C). The results show that the expression levels of *GAME2*, *GAME5*, and *GAME18* were higher in the *E8: SlERF.D6* lines compared with the EV-E8 line, whereas the expression levels of *GAME12* were lower (Figure 5B and Supplementary Table 8). In *TRV2: SlERF.D6* lines, the expression levels of *GAME2*, *GAME5*, and *GAME18* were lower and the expression levels of *GAME12* were higher (Figure 5C). Additionally, we measured the concentration of SGA biosynthesis-related metabolites using liquid chromatography–mass spectrometry (LC-MS). The data showed that the levels of furostanol-26-aldehyde, β1-tomatine, α-tomatine, and esculeoside A were significantly higher in the *E8: SlERF.D6* lines compared with the EV-E8 line, whereas the levels of tomatidine were lower (Figure 5D and Supplementary Table 9). Perhaps, the opposite was true in the *TRV2: SlERF.D6* lines (Figure 5E). It is noteworthy that the level of esculeoside A was at least fivefold lower in the fruit from the *TRV2: SlERF.D6* lines relative to the fruit from the EV-TRV2 line, which was significant than other metabolites (Figure 5E).

To investigate whether SlERF.D6 binds to the promoters of *GAME* genes to directly modulate SGA metabolism in tomato fruit ripening, a yeast one-hybrid (Y1H) assay was performed. The results showed that yeast containing the combination of SlERF.D6 and *GAME12pro* can grow normally on −Leu/Trp/His medium, and the control yeast contained the combination of pGADT7 empty vector and *GAME12pro* showed the opposite result. However, there was no similar result on the other three genes (Supplementary Figure 10A), which means that SlERF.D6 binds to the promoter of *GAME12*, but not to *GAME2*, *GAME5*, and *GAME18*. To further confirm the results, we performed a dual-luciferase experiment in tobacco leaves. The dual-luciferase analysis showed that co-expression with *SlERF.D6* significantly decreased the luciferase activity of *GAME12pro* but had no effect on other *GAME* genes (Supplementary Figure 10B). These results suggest that SlERF.D6, acting as a transcriptional repressor, negatively regulates the synthesis of tomatidine *via* the direct transcriptional repression of *GAME12* and it positively regulates the catabolism of tomatidine *via* the indirect transcriptional promotion of other three genes.

## Discussion

Transcription factors, such as the NAC, MYB, bHLH, WRKY, and AP2/ERF families, are widely involved in the fruit development and metabolic regulation in plants (Groszmann et al., 2008; Machemer et al., 2011; Liu et al., 2018). In recent years, there are numerous reports on the identification and analysis of AP2/ERF gene families in plants, such as tomato, rice, rape, and other plants. They can regulate fruit ripening and respond to a variety of stressful environments (Du et al., 2016; Klay et al., 2018; Donde et al., 2019; Song et al., 2020; Shoji and Yuan, 2021). With the development of sequencing technology, more and more plant genome data are reassembled to obtain higher quality genome (Jung et al., 2019; Bohra et al., 2020), and the research of AP2/ERF family is more and more in-depth and comprehensive. Thus, identifying AP2/ERF TFs can considerably improve our understanding about their evolution and function in various plant species. A total of *AP2/ERF* genes in the tomato genome resulted in the identification of 176 members, and previous studies have revealed that with the AP2/ERF TFs, the number was 146 and we expanded the number of this family and given the basic characterization (Pirrello et al., 2012). In addition, we found 45 of them under domestication or improvement stage, it suggested that these genes may regulate some traits that were relevant to humans.

Tomatidine that determine the bitterness are gradually synthesized into esculeoside A accumulated in red mature fruits (Nakayasu et al., 2018). We performed mGWAS to scan the gene *SlERF.D6* associated with esculeoside A (Figure 3A). Next, we found during fruit development, the content of tomatidine decreased rapidly, while esculeoside A content increased significantly (Figures 5D,E), which was consistent with previous studies (Cárdenas et al., 2016). In addition, the ERF.D6 presented in the group which might be the main factor for the change of fruit color and firmness, and genetically modified breeding will be generated by genetic engineering technology to improve the ripening time and nutritional quality of tomato in future. Therefore, this study conjectured that *SlERF.D6* could regulate SGA metabolism and fruit development in addition to the functions of resisting abiotic stress (Li et al., 2018).

Natural variation in *ERF.D6* can disrupt the normal metabolic levels of SGAs, and we also found it under the domestication or improvement selection (Figure 3E), it showed that *ERF.D6* underwent natural variation in the population and the variation in this gene was associated with esculeoside A, and this different trait was selected by ancient farmers. In addition, the natural variation in *ERF.D6* can promote fruit ripening, allowing farmers to obtain ripe tomato fruit faster and shorten the growing cycle. Thus, the candidate gene *ERF.D6* both regulates fruit development and at the same time is an important gene for altering the nutritional varieties of tomato (reducing toxicity). Selection for haplotype of *ERF.D6* and rational crosses could lead to new tomato varieties with faster fruit ripening and less antinutritional metabolites, which could be of great use for tomato breeding applications and crop genetic improvement.

Plant growth and development are coordinated by a complex network of interacting phytohormones, and the interplay between ethylene and auxin signaling is critical for a wide range of plant developmental processes. In a previous study, Liu et al. (2018) demonstrated the role of tomato *ERF.B3* in integrating ethylene and auxin signaling by directly regulating *Sl-Aus/IAA27*, while Wang et al. (2009) commented on the role of Aux/IAA in plant specialized metabolism. In another work, it showed that the reciprocal *AP2/ERF* gene clusters regulate the biosynthesis of specific metabolites in a variety of plants (Paul et al., 2020), such as *ORCA5* overexpression in tobacco and *NICOTINE2 ERF189* overexpression in *Catharanthus roseus* activate nicotine and terpenoid indole alkaloid pathway genes, respectively (Kato et al., 2014). In SGAs biosynthesis, studies shown that phytohormones, such as JA and GA, can regulate SGAs to resolve chemical defense with growth in tomato (Nakayasu et al., 2018; Panda et al., 2022). In view of the above research content, we speculate that *SlERF.D6* may be induced by phytohormones to regulate SGAs.

## Data Availability Statement

The datasets presented in this study can be found in online repositories. The names of the repository/repositories and accession number(s) can be found below: National Center for Biotechnology Information (NCBI) BioProject database under accession number PRJNA783378.

## Author Contributions

SW conceived the project and supervised the study. HG and YD conducted the data analysis and curation. MM and PC contributed to experimentation and methodology. RC and JL performed the metabolite profiling. YZ, LS, CW, CL, YL, QB, and TT participated in the material preparation. HG, MM, and PC wrote the first draft. SW and JY reviewed, edited, and finalized the manuscript. All authors discussed the results and commented on the manuscript.

## Conflict of Interest

The authors declare that the research was conducted in the absence of any commercial or financial relationships that could be construed as a potential conflict of interest.

## Publisher’s Note

All claims expressed in this article are solely those of the authors and do not necessarily represent those of their affiliated organizations, or those of the publisher, the editors and the reviewers. Any product that may be evaluated in this article, or claim that may be made by its manufacturer, is not guaranteed or endorsed by the publisher.

## Footnotes

1
https://solgenomics.net/

2
http://pfam.xfam.org/

3
http://smart.embl-heidelberg.de/

4
http://web.expasy.org/protparam/

5
https://www.genscript.com/wolf-psort.html

6
http://www.vardb.org/vardb/analysis/gblocks.html

7
ftp://ftp.ensemblgenomes.org/

8
http://www.cog-genomics.org/plink
