# Supplementary material for: Multi-Omics Analysis Reveals That SlERF.D6 Synergistically Regulates SGAs and Fruit Development
Source: Front Plant Sci. 2022 Apr 8;13:860577. doi: 10.3389/fpls.2022.860577 (PMC9024245; doi:10.3389/fpls.2022.860577)
Supplement: Supplementary file 1 [file Presentation_1.pdf]

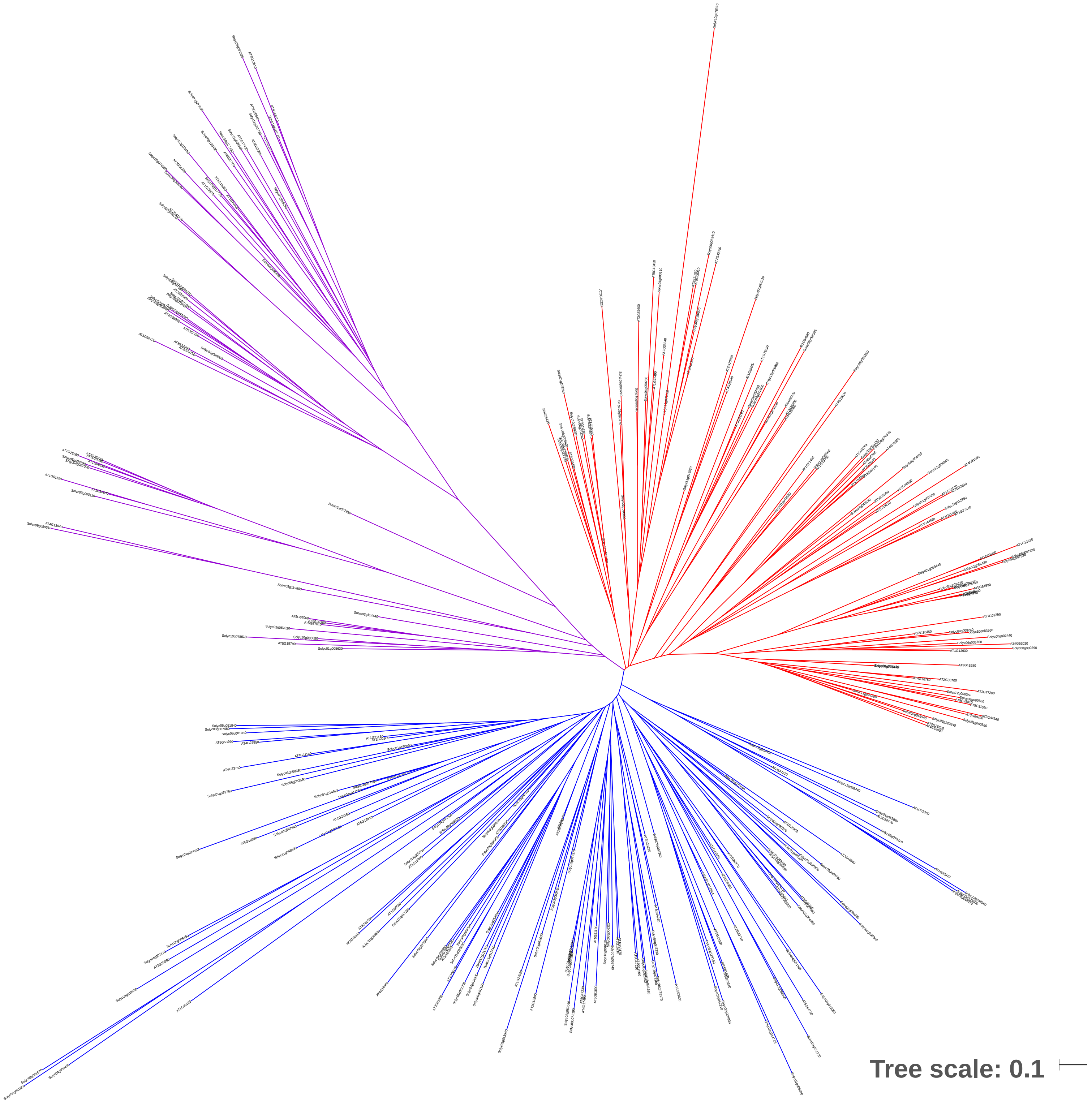

Supplementary Figure 1. Phylogenetic analysis of all AP2/ERF family genes in tomato and Arabidopsis. Red represents AP2 subfamily, blue represents ERF subfamily, and purple represents RAV subfamily.The genes ID was labeled at each branch.

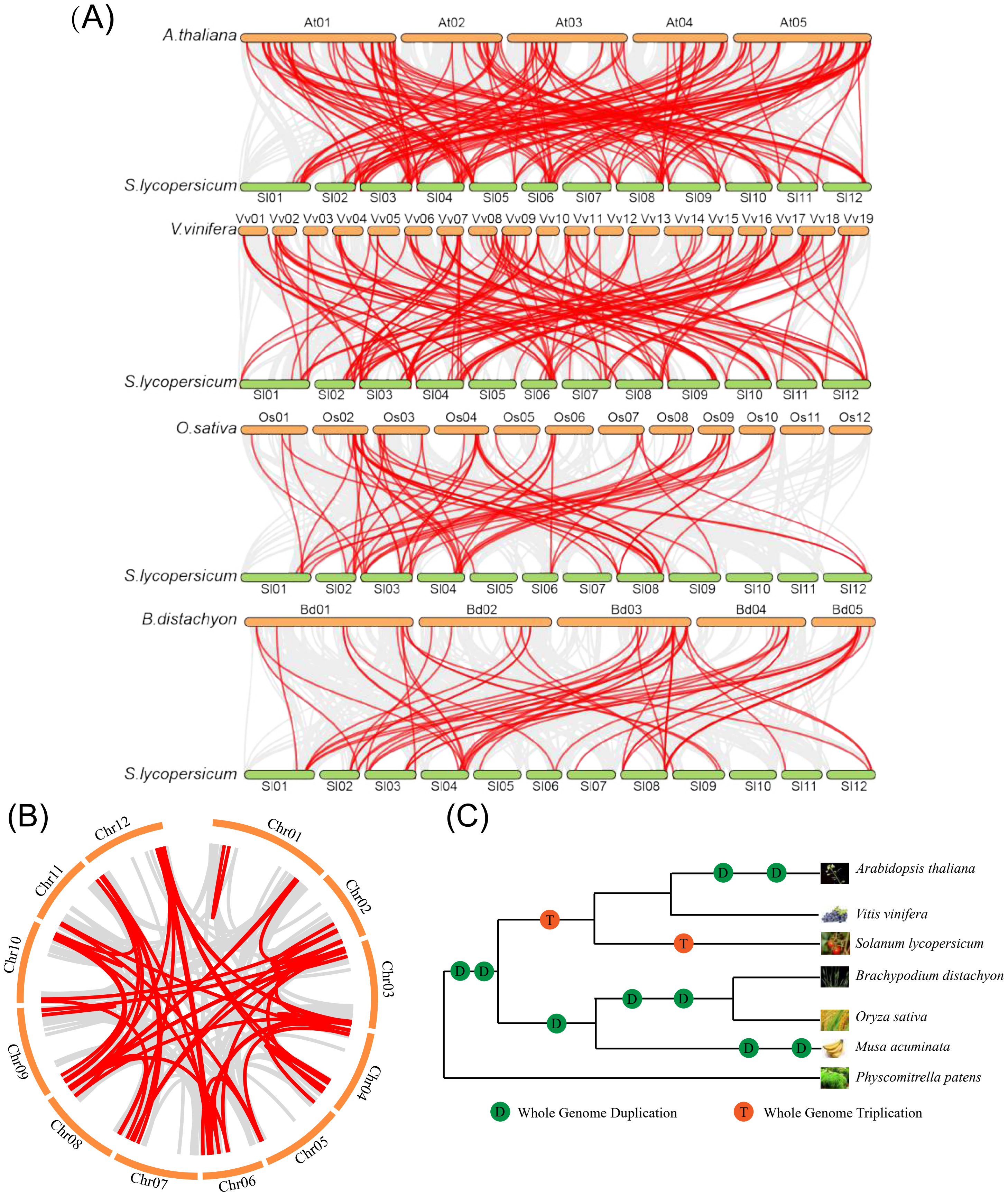

Supplementary Figure 2. Evolutionary and Synteny analysis of AP2/ERF genes between tomato and four representative plant species. (A) Synteny between Solanum lycopersicum and other four plants (Arabidopsis thaliana, Vitis vinifera, Oryza sativa and Brachypodium distachyon). Gray lines in the background indicate the collinear blocks within tomato and other plant genomes, while the red lines highlight the syntenic AP2/ERF gene pairs. (B) Distribution of Solanum lycopersicum genomic features. Gray lines indicate all synteny blocks in the tomato genome, and the red lines indicate duplicated AP2/ERF gene pairs. (C) Hypothetical scheme of evolutional events in AP2/ERF gene family among monocot and eudicot plants. The timing of whole-genome duplication (WGD) and the timing of whole-genome triplication (WGT) are superimposed on the tree.

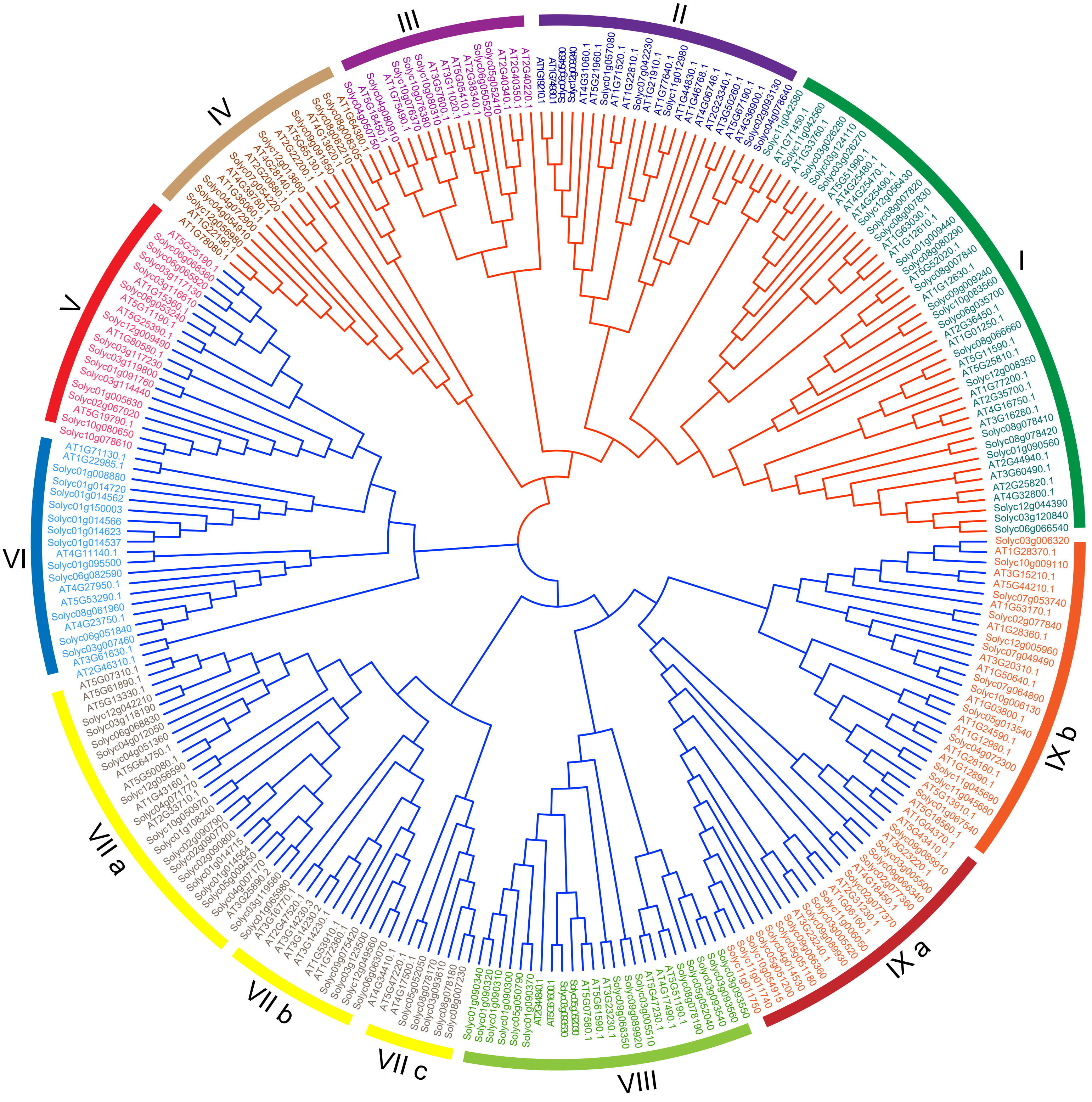

Supplementary Figure 3. Phylogenetic tree of ERF proteins from tomato and *Arabidopsis thaliana*. Each color of the tips and the letters of the outer ring represent a different branch. Red and blue branches represent DREB and ERF subfamilies.

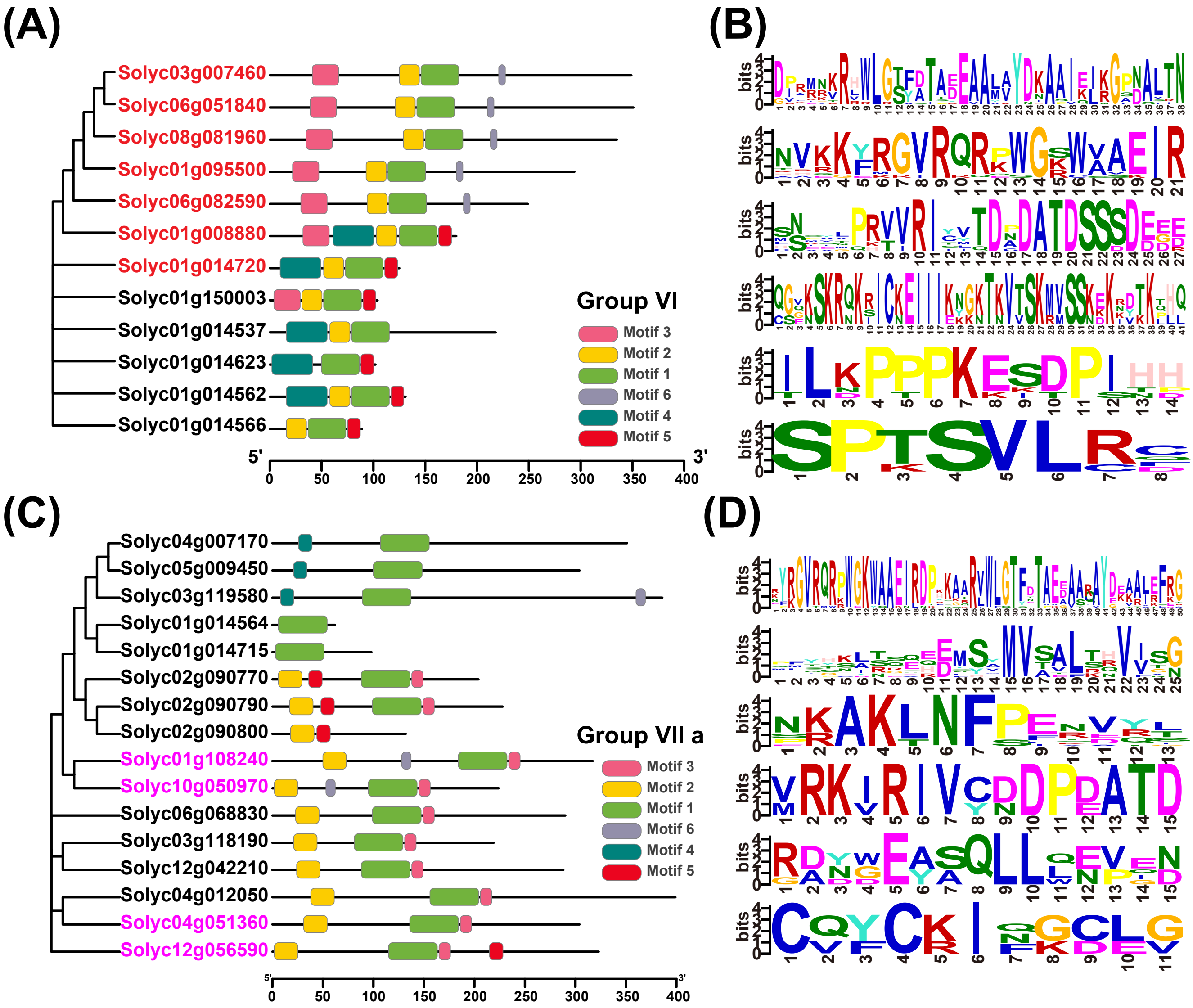

Supplementary Figure 4. The analysis of conserved motif among some ERF groups. The gene with red and purple color represent they are associated with ethylene and cytokinin. The x-axis of A and C is length of nucleotides, and th x-axis of B and D is the location of the base in the motif.

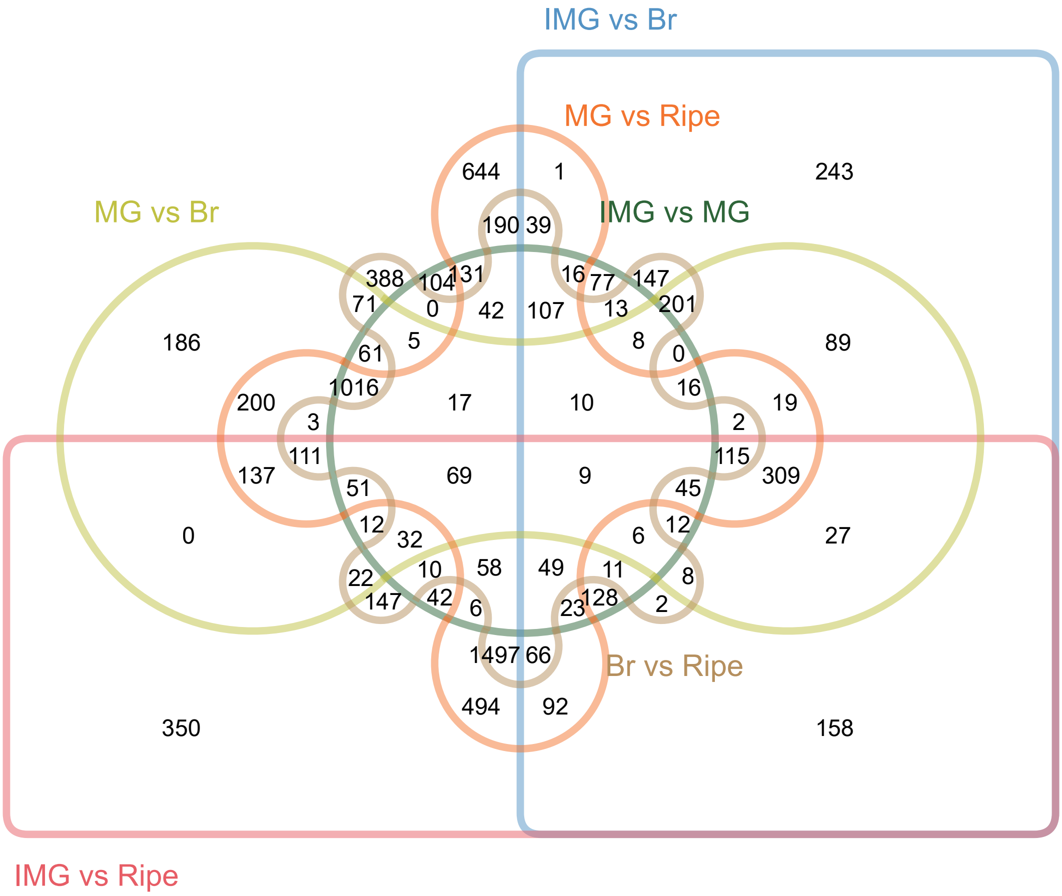

Supplementary Figure 5. Comparison of differentially expressed genes (DEGs) among multiple fruit development stages. The number represents how many DEGs in different comparison.

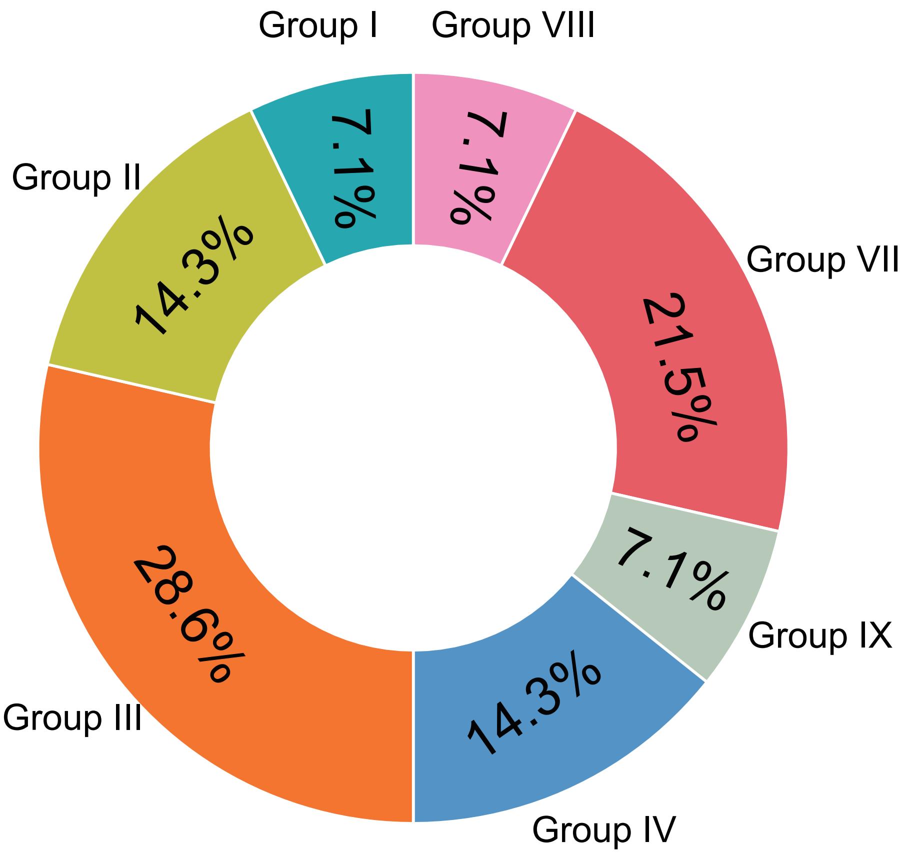

Supplementary Figure 6. The pie plot shows the classification of ERF genes that may promote fruit ripening. The numbers represent the percentage of genes in these groups among all candidate genes.

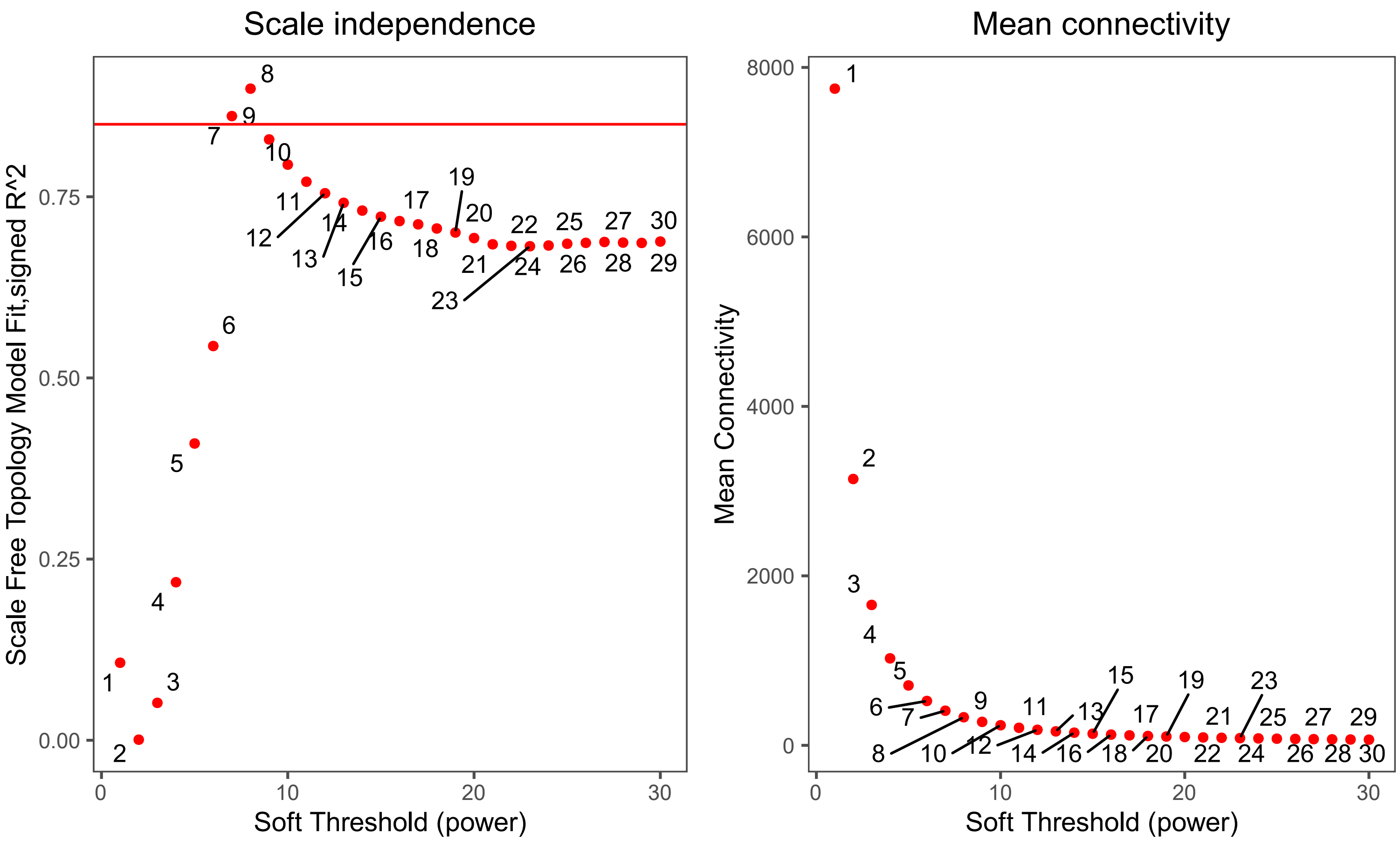

Supplementary Figure 7. The parameter, soft threshold, determination for module construction. The best value is 7 for this dataset. The x-axis is the candidate soft thresholds, the y-axis of the left figure is the R^2 of scale-free network with different soft threshold, the right one is the mean connectivity with different soft threshold.

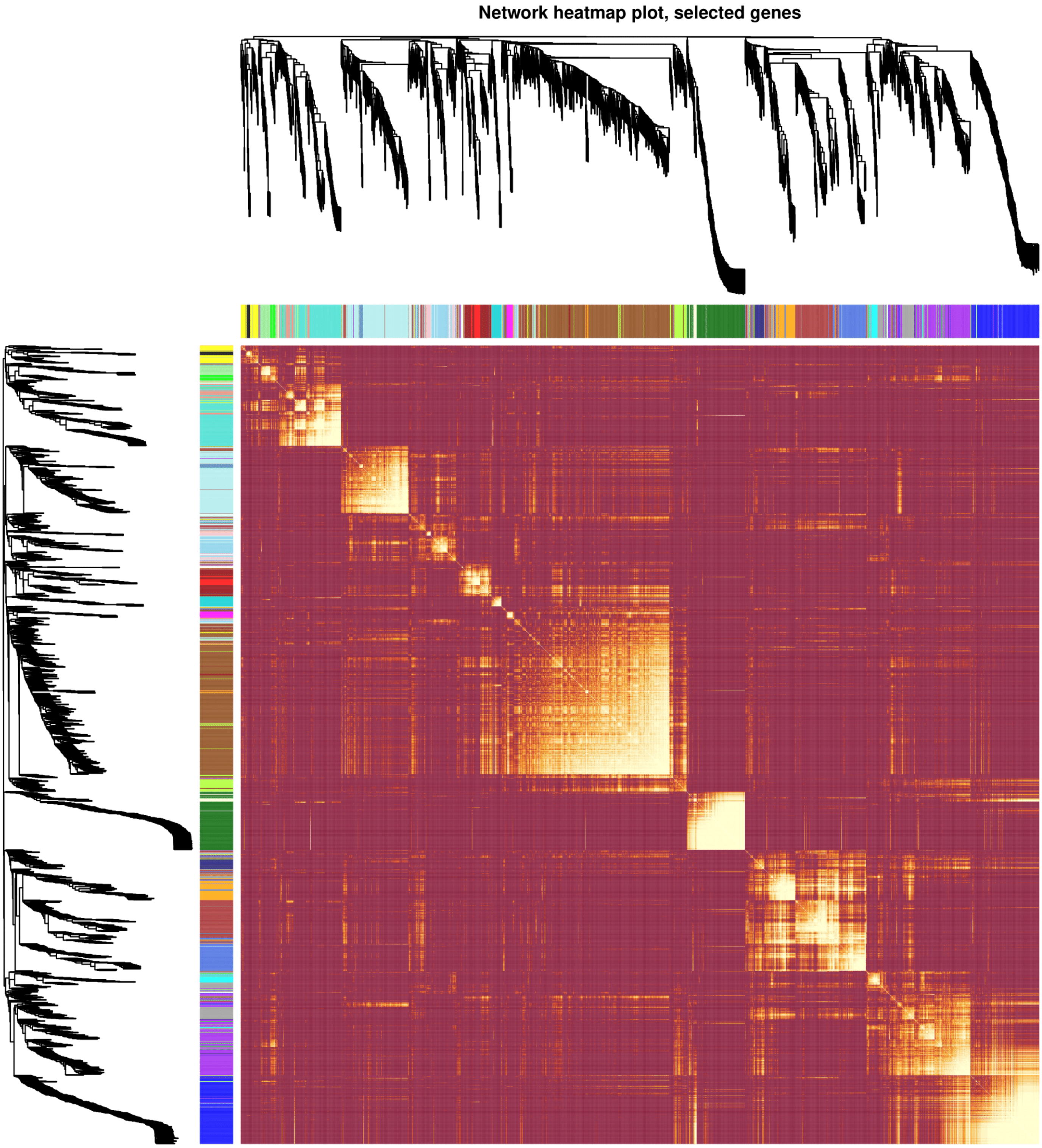

Supplementary Figure 8. The TOM plot of WGCNA. Each point on the x-axis and y-axis represents one gene, and the color of the heatmap indicates the correlation between genes at the transcriptional level.

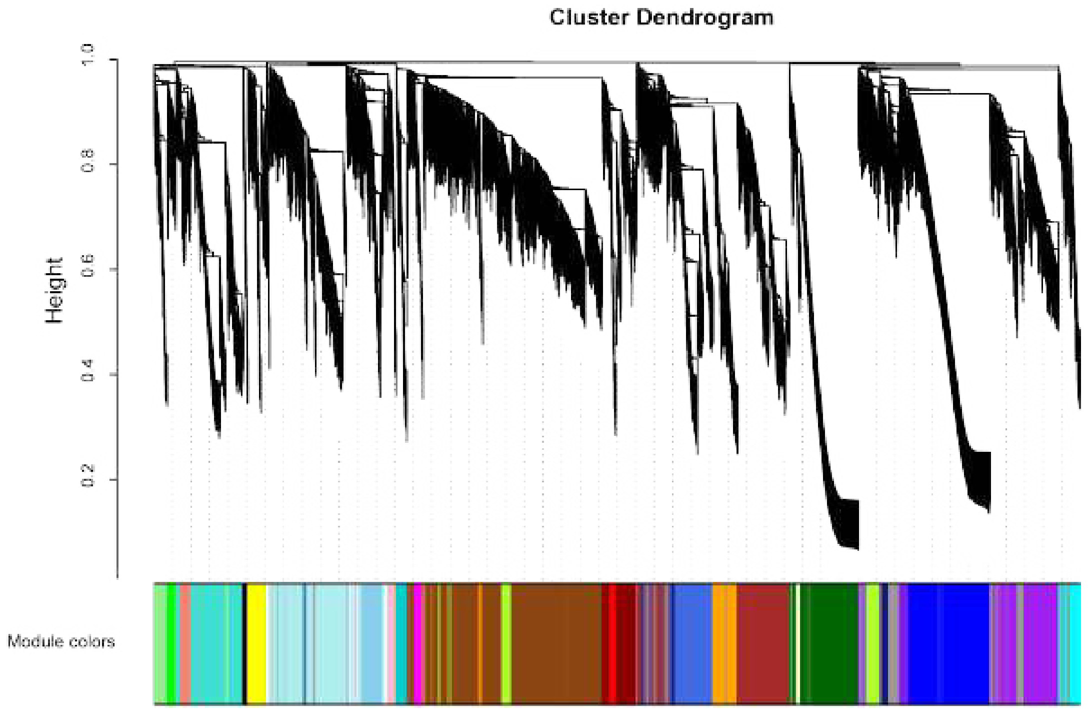

Supplementary Figure 9. Hierarchical clustering tree (dendrogram) of genes based on co-expression network analysis. Each branch represents one gene, and each color is a model was identified by WGCNA.

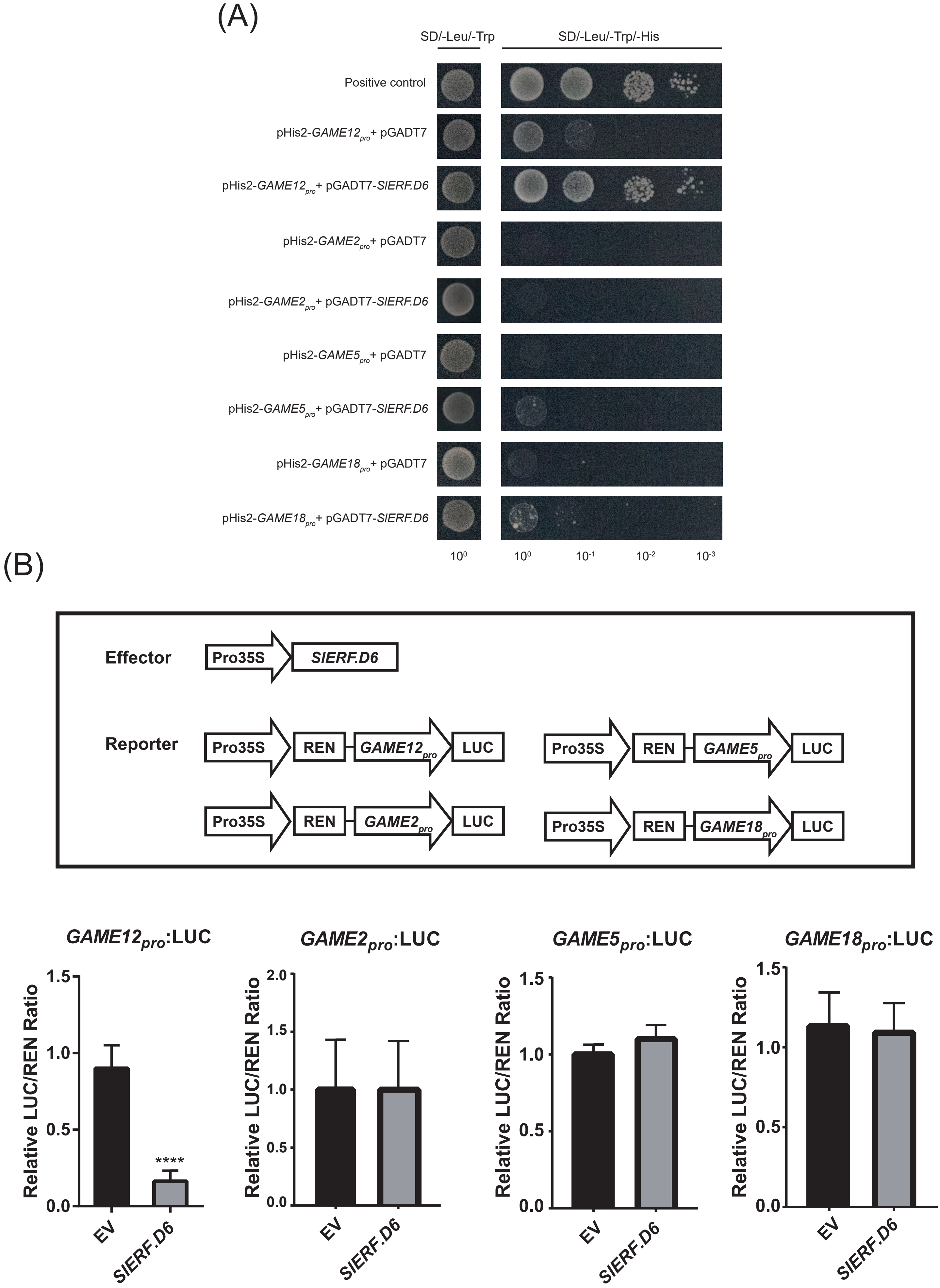

Supplementary Figure 10. *GAME* genes promoter-binding activity of SlERF.D6 (A) and transactivation activity of SlERF.D6 on *GAME* genes promoter in *Nicotiana benthamiana* leaves (B).

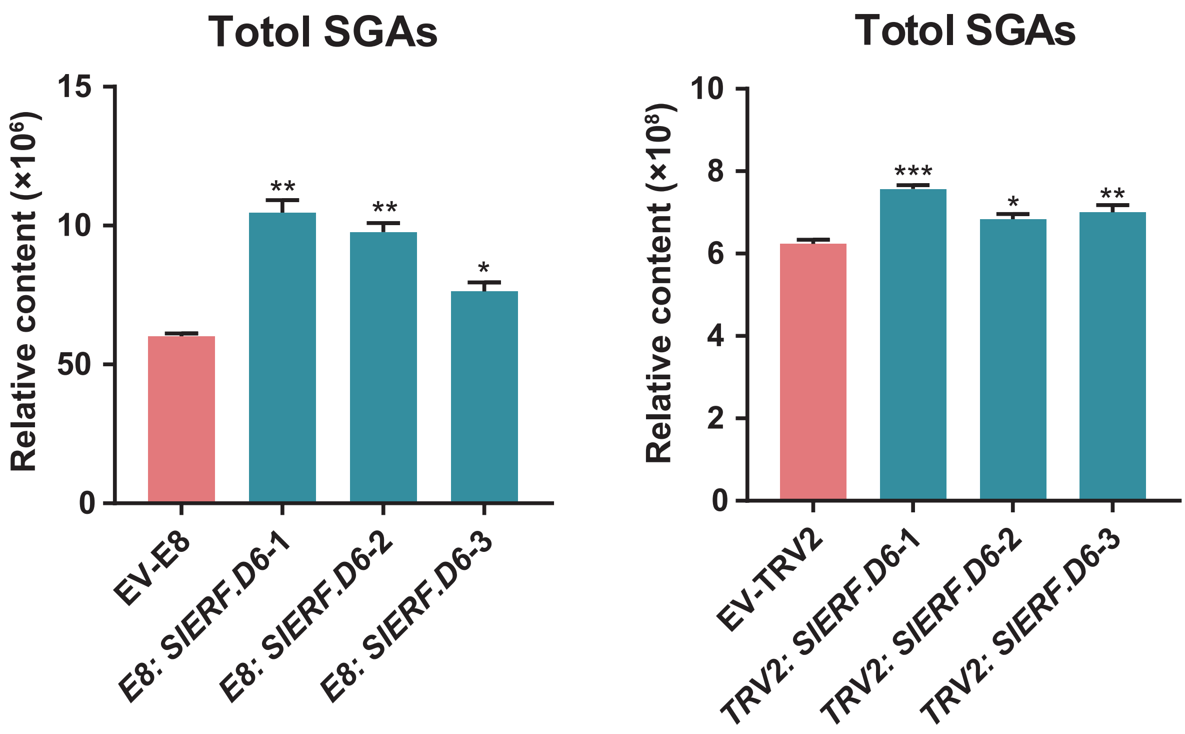

Supplementary Figure 11. The contents of total SGAs in *E8: SlERF.D6* and *TRV2: SlERF.D6* lines. The Y axis represents relative content. All the above error bars represent the SD (n =3) (*P < 0.05, **P < 0.01; Student's t-test).
